# Influence of sodium/glucose cotransporter-2 inhibitors on the incidence of acute kidney injury: a meta-analysis

**DOI:** 10.3389/fphar.2024.1372421

**Published:** 2024-06-25

**Authors:** Qian Wang, Jianmin Yu, Weizhu Deng, Chao Liu, Jian Yang, Yaqing Li, Guangyan Cai, Xiangmei Chen, Zheyi Dong

**Affiliations:** ^1^ Department of Nephrology, First Medical Center of Chinese PLA General Hospital, Nephrology Institute of the Chinese People’s Liberation Army, National Key Laboratory of Kidney Diseases, National Clinical Research Center for Kidney Diseases, Beijing Key Laboratory of Kidney Disease Research, Beijing, China; ^2^ Department of Diagnosis and Treatment, The Eighth Medical Center of Chinese PLA General Hospital, Beijing, China; ^3^ Department of Critical Care Medicine, The First Medical Center of Chinese PLA General Hospital, Beijing, China

**Keywords:** acute kidney injury, meta-analysis, randomized controlled trials, sodium/glucose cotransporter-2 inhibitors, systematic review

## Abstract

**Background:**

Sodium/glucose cotransporter-2 inhibitors (SGLT2i) are associated with cardiovascular benefits. The aim of this systematic review and meta-analysis is to summarize the influence of SGLT2i on the incidence of acute kidney injury (AKI), and to ascertain whether it is affected by confounding variables such as age, baseline renal function and concurrent use of renin-angiotensin-aldosterone system inhibitors (RAASi) or mineralocorticoid receptor antagonists (MRA).

**Methods:**

PubMed, Embase, and Cochrane Library databases were searched for randomized controlled trials comparing the influence of SGLT2i versus placebo/blank treatment on AKI in the adult population. A fixed-effect model was used if the heterogeneity was not significant; otherwise, a randomized-effect model was used.

**Results:**

Eighteen studies comprising 98,989 patients were included. Compared with placebo/blank treatment, treatment with SGLT2i significantly reduced the risk of AKI (risk ratio [RR]: 0.78, 95% confidence interval [CI]: 0.71 to 0.84, *p* < 0.001; *I* ^2^ = 0%). Subgroup analysis suggested consistent results in patients with diabetes, chronic kidney disease, and heart failure (for subgroup difference, *p* = 0.32). Finally, univariate meta-regression suggested that the influence of SGLT2i on the risk of AKI was not significantly modified by variables such as age (coefficient: 0.011, *p* = 0.39), baseline estimated glomerular filtration rate (coefficient: −0.0042, *p* = 0.13) or concomitant use of RAASi (coefficient: 0.0041, *p* = 0.49) or MRA (coefficient: −0.0020, *p* = 0.34).

**Conclusion:**

SGLT2i may be effective in reducing the risk of AKI, and the effect might not be modified by age, baseline renal function and concurrent use of RAASi or MRA.

## Introduction

Sodium/glucose cotransporter-2 inhibitors (SGLT2i) represent a novel class of oral antidiabetic medications that have demonstrated additional advantageous effects on cardiac and renal function (Frak et al., 2023; Klen and Dolzan, 2023; Lam-Chung, 2023). From a pharmacological standpoint, SGLT2i functions by inhibiting the reabsorption of glucose in the initial proximal tubule of the kidney, thereby augmenting the excretion of glucose in the urine and reducing the overall glucose burden on the body (Vallon and Verma, 2021). In individuals diagnosed with type 2 diabetes mellitus (T2DM), an initial meta-analysis of three extensive clinical trials revealed that the utilization of SGLT2i was associated with an 11% decrease in the risk of major adverse cardiovascular events, a 23% decrease in the risk of cardiovascular death or hospitalization for heart failure (HF), and a 45% decrease in the risk of progression of renal disease (Zelniker et al., 2019). In a study involving patients with HF, it was demonstrated that SGLT2i effectively reduced the likelihood of cardiovascular death and hospitalizations for HF across a diverse range of patients, thus establishing their significance as a fundamental therapy for HF, regardless of ejection fraction or care setting (Vaduganathan et al., 2022). Furthermore, a recent meta-analysis encompassing 13 clinical trials revealed that SGLT2i exhibited efficacy in altering the risk of kidney disease progression, not only in patients with T2DM at high cardiovascular risk, but also in patients with chronic kidney disease (CKD) or HF regardless of diabetic status (2022). Consequently, the indications for SGLT2i have expanded beyond T2DM to include HF and CKD, supported by accumulating evidence. Nevertheless, conflicting findings have emerged regarding the potential occurrence of acute kidney injury (AKI) when utilizing SGLT2i (Copur et al., 2023). One case report documented a dialysis-dependent AKI following the initiation of SGLT2i, with a suggested association to osmotic nephropathy (Phadke et al., 2020). Furthermore, a recent investigation utilizing the most up-to-date records from the United States Food and Drug Administration’s Adverse Event Reporting System has indicated a potential link between SGLT2i and the development of AKI, although this association may be mitigated in instances where renin-angiotensin-aldosterone system inhibitors (RAASi), such as angiotensin converting enzyme inhibitors (ACEI) or angiotensin II receptor blockers (ARB), are concurrently administered (Katsuhara and Ikeda, 2021). Nevertheless, it is worth noting that several observational studies have failed to demonstrate an elevated risk of AKI associated with the use of SGLT2 inhibitors (Rampersad et al., 2020; Zhuo et al., 2022). In order to conduct a comprehensive assessment of the impact of SGLT2i on the occurrence of AKI, we conducted a systematic review and meta-analysis of eligible randomized controlled trials (RCTs). Furthermore, we investigated whether the effect of SGLT2i on the risk of AKI could be influenced by study-specific factors such as age, baseline renal function, and concurrent use of RAASi at the study level.

## Methods

This study was designed and implemented according to the Cochrane Handbook guidelines (Higgins et al., 2021) and the PRISMA (Preferred Reporting Items for Systematic Reviews and Meta-Analyses) statement (Page et al., 2021a; Page et al., 2021b).

### Search strategy

A combination of strategies was used to search PubMed, Embase, and Cochrane Library for relevant studies with: (1) “SGLT 2 inhibitor” OR “SGLT-2 inhibitor” OR “SGLT2” OR “sodium glucose transporter 2 inhibitor” OR “sodium glucose transporter ii inhibitor” OR “sodium glucose cotransporter 2 inhibitors” OR “dapagliflozin” OR “canagliflozin” OR “tofogliflozin” OR “bexagliflozin” OR “empagliflozin” OR “luseogliflozin” OR “remogliflozin” OR “ertugliflozin” OR “henagliflozin” OR “ipragliflozin” OR “licogliflozin” OR “sergliflozin” OR “sotagliflozin”; (2) “acute” OR “abrupt”; (3) “kidney” OR “renal”; and (4) “random” OR “randomly” OR “randomized” OR “control” OR “placebo”. Relevant clinical studies have been limited to humans. We also manually searched for reference lists to review and original articles that were related to the topic. Database searches were conducted on 14 May 2024.

### Study selection

Studies were included if they fulfilled the following criteria according to the PICOS principles.

P (patients): adult patient population without limitations of the diagnosis, which could be patients with T2DM, CKD, or HF.

I (intervention): SGLT2i.

C (control): placebo or blank treatment.

O (outcome): incidence of AKI compared between patients with SGLT2i and controls during follow-up. The diagnosis of AKI was in accordance with the criteria used among the original studies

S (study design): parallel-group RCTs, published as full-length articles in peer-reviewed journals.

Non-RCTs, studies that did not include an intervention group of SGLT2i, those comparing the effects of different doses of SGLT2i, single-arm studies without controls, or studies not evaluating the outcome of AKI were excluded. For studies with overlapping patients, the study with the largest sample size was included.

### Data extraction and quality assessment

The process of data extraction, mining, and quality evaluations was carried out by two authors working independently. In the event of any disagreement, the corresponding author was consulted to address and resolve such inconsistencies. Information regarding publication details (author, year of publication, and study country), study design (blind or open-label), patient characteristics (diagnosis, demographic information, baseline renal function as evaluated by estimated glomerular filtration rate (eGFR), proportions of patients with concurrent use of any RAASi, and proportions of patients with concurrent use of mineralocorticoid receptor antagonists [MRA]), details of interventions and controls, follow-up durations, and diagnostic criteria for AKI was extracted. The quality of RCTs was assessed utilizing the Cochrane Risk of Bias Tool (Higgins et al., 2021), adhering to the subsequent criteria: (1) random sequence generation; (2) allocation concealment; (3) participant and staff blinding; (4) outcome assessor blinding; (5) presentation of incomplete outcome data; (6) reporting of selective results; and (7) identification of other potential biases.

### Statistical analysis

The numbers of patients with AKI events and total numbers of patients allocated to the SGLT2i and control groups were extracted from the original reports. The influence of SGLT2i on the incidence of AKI in adult patients compared to control was summarized as risk ratio (RR) and corresponding 95% confidence intervals (CIs). The Cochrane Q test was performed (Higgins and Thompson, 2002). Heterogeneity was also estimated by calculating *I* ^2^ and *I* ^2^ > 50% suggested significant heterogeneity (Higgins et al., 2003). In the pooled analyses, a random-effects model was employed when significant heterogeneity was identified; alternatively, a fixed-effects model was utilized (Higgins et al., 2021). A sensitivity analysis was performed by only including high-quality studies (all seven domains of Cochrane Risk of Bias Tool judged as low risk). Additionally, a predefined subgroup analysis was conducted based on the patients’ diagnosis and the specific SGLT2i drugs administered. Furthermore, a univariate meta-regression analysis was conducted to investigate whether the study characteristics of continuous variables could significantly alter the impact of SGLT2i on AKI, such as mean age of the patients, proportion of men, mean eGFR at baseline, proportion of patients using any RAASi, proportion of patients using MRA, and mean follow-up duration of the study. Publication bias was assessed using funnel plots and Egger’s regression asymmetry test (Egger et al., 1997). Statistical significance was defined as *p* < 0.05. The statistical analysis was performed using Stata software (version 12.0; Stata Corporation) and RevMan (version 5.1; Cochrane, Oxford, United Kingdom).

## Results

### Search results

**FIGURE 1:**
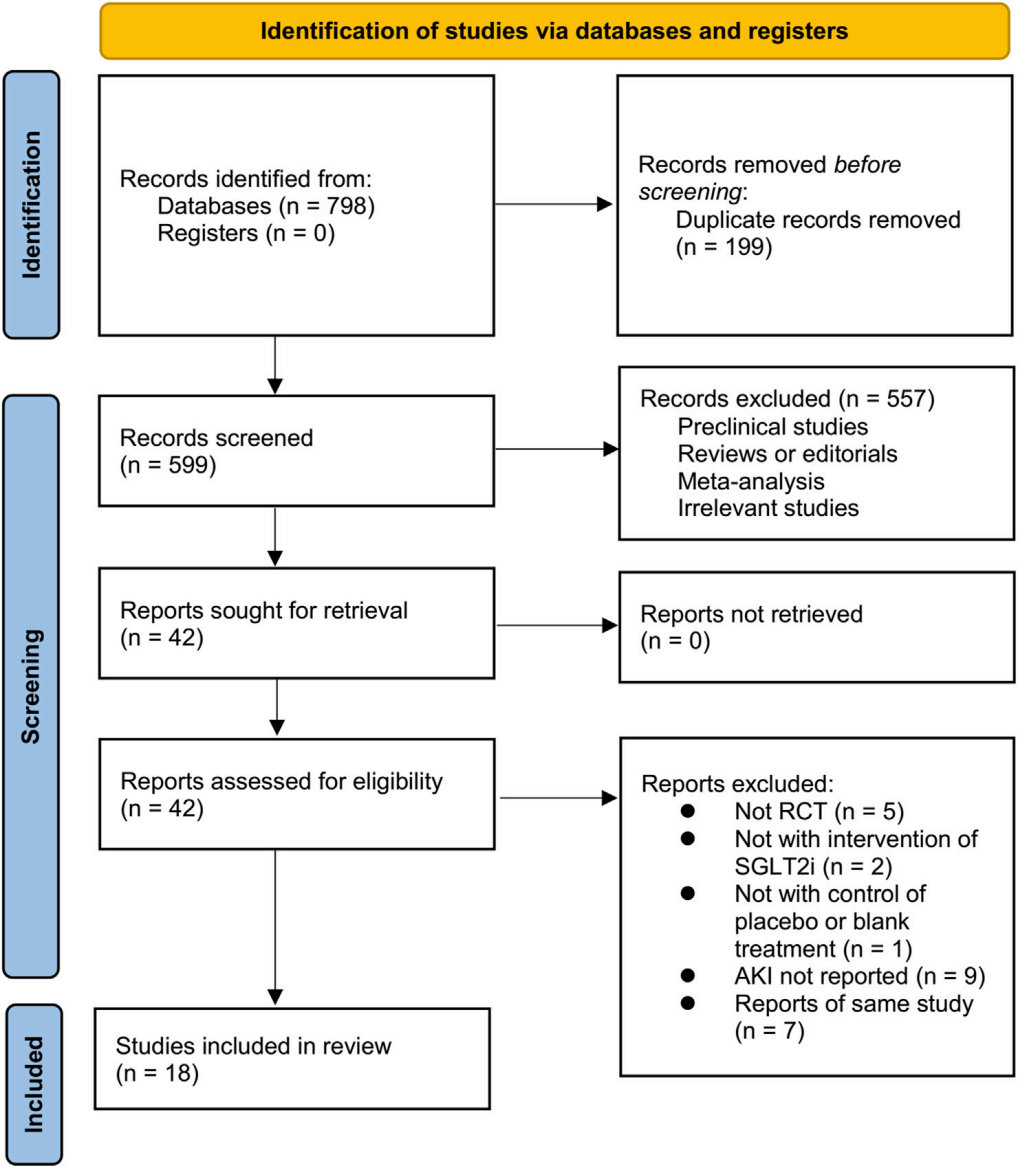
Flowchart of literature search.

A diagram illustrating the process of database searching and study identification is presented in Figure 1. The search of the databases yielded a total of 798 articles, of which 599 were identified as unique after removing duplicates. Subsequently, 557 articles were excluded based on their title and abstract, primarily due to their lack of relevance to the research objectives. A thorough examination of the full text was conducted on 42 articles, resulting in the exclusion of 24 articles for the reasons depicted in Figure 1. Ultimately, the final analysis encompassed a total of 18 RCTs (Zinman et al., 2015; Neal et al., 2017; McMurray et al., 2019; Perkovic et al., 2019; Wiviott et al., 2019; Cannon et al., 2020; Heerspink et al., 2020; Bhatt et al., 2021a; Anker et al., 2021; Bhatt et al., 2021b; Kosiborod et al., 2021; Zannad et al., 2021; Solomon et al., 2022; Voors et al., 2022; Feitosa et al., 2023; Herrington et al., 2023; Butler et al., 2024; Cox et al., 2024).

### Study characteristics and data quality

An overview of the included studies is presented in Table 1. Since one of the included studies reported the outcome according to different doses of SGLT2i, and the other one reported the outcome according to whether the patients were with CKD, these datasets were included independently in the meta-analysis. Overall, 20 datasets from 18 RCTs (Zinman et al., 2015; Neal et al., 2017; McMurray et al., 2019; Perkovic et al., 2019; Wiviott et al., 2019; Cannon et al., 2020; Heerspink et al., 2020; Bhatt et al., 2021a; Anker et al., 2021; Bhatt et al., 2021b; Kosiborod et al., 2021; Zannad et al., 2021; Solomon et al., 2022; Voors et al., 2022; Feitosa et al., 2023; Herrington et al., 2023; Butler et al., 2024; Cox et al., 2024) involving 98,989 patients were included. Generally, patients with T2DM, CKD, HF, acute myocardial infarction, and hospitalized patients with COVID-19 were included. The mean ages of the patients were 61–72 years, with the baseline mean eGFR varying from 37 to 85 mL/min/1.73 m^2^. In the intervention group, SGLT2i including empagliflozin, canagliflozin, dapagliflozin, ertugliflozin, and sotagliflozin were used. The follow-up durations were from 1 to 50 months. As for the diagnosis for AKI, the Medical Dictionary for Regulatory Activities (MDRA) preferred term for AKI was used for most of the included studies (Zinman et al., 2015; Neal et al., 2017; McMurray et al., 2019; Perkovic et al., 2019; Wiviott et al., 2019; Cannon et al., 2020; Bhatt et al., 2021a; Anker et al., 2021; Bhatt et al., 2021b; Kosiborod et al., 2021; Zannad et al., 2021; Solomon et al., 2022; Voors et al., 2022; Butler et al., 2024; Cox et al., 2024), while for the other studies, a doubling (Heerspink et al., 2020) or a 1.5-times increment of serum creatinine (Herrington et al., 2023) or the Kidney Disease Improving Global Outcomes criteria (Feitosa et al., 2023) were used. According to Table 2, the quality of each included RCTs was assessed according to the Cochrane Risk of Bias Tool. Most of the included studies were double-blind placebo controlled studies (Zinman et al., 2015; Neal et al., 2017; McMurray et al., 2019; Perkovic et al., 2019; Wiviott et al., 2019; Cannon et al., 2020; Heerspink et al., 2020; Bhatt et al., 2021a; Anker et al., 2021; Bhatt et al., 2021b; Kosiborod et al., 2021; Zannad et al., 2021; Solomon et al., 2022; Voors et al., 2022; Herrington et al., 2023; Butler et al., 2024) with adequate report of details of random sequence generation and allocation concealment. Only two studies were open-label studies (Feitosa et al., 2023; Cox et al., 2024), with no detailed report of random sequence generation or allocation concealment.

**TABLE 1:** Characteristics of the included studies.

Study	Design	Patient diagnosis	No of patients	Mean age (years)	Men (%)	Baseline eGFR (ml/min/1.73 m^2^)	Any RAASi (%)	MRA (%)	Intervention	Control	Follow-up duration (months)	Diagnosis of AKI
Zinman et al. (2015) 10 mg	R, DB, PC	T2DM patients at high CV risk	3,512	63.1	71	74.1	80.6	6.4	Empagliflozin 10 mg/d	Placebo	37.2	MedDRA Preferred Term for AKI
Zinman et al. (2015) 25 mg	R, DB, PC	T2DM patients at high CV risk	3,508	63.2	72	73.9	80.8	6.2	Empagliflozin 25 mg/d	Placebo	37.2	MedDRA Preferred Term for AKI
Neal et al. (2017)	R, DB, PC	T2DM patients at high CV risk	10,142	63.3	64.2	76.5	80	NR	Canagliflozin 100 or 300 mg/d	Placebo	47.1	MedDRA Preferred Term for AKI
Perkovic et al. (2019)	R, DB, PC	T2DM patients with albuminuric CKD	4,401	63	66.1	56.2	99.9	NR	Canagliflozin 100 mg/d	Placebo	31.4	MedDRA Preferred Term for AKI
McMurray et al. (2019)	R, DB, PC	Patients with HFrEF	4,744	66.4	76.6	65.7	94.4	71.1	Dapagliflozin 10 mg/d	Placebo	18.2	MedDRA Preferred Term for AKI
Wiviott et al. (2019)	R, DB, PC	T2DM patients who had or were at risk for ASCVD	17,160	64	62.6	85.2	81.3	NR	Dapagliflozin 10 mg/d	Placebo	50.4	MedDRA Preferred Term for AKI
Heerspink et al. (2020)	R, DB, PC	Patients with eGFR of 25–75 mL/min/1.73 m^2^	4,304	61.9	66.9	43.1	98.1	NR	Dapagliflozin 10 mg/d	Placebo	28.8	A doubling of SCr compared with most recent results
Cannon et al. (2020)	R, DB, PC	T2DM patients with ASCVD	8,246	64.4	69.9	75.9	80.1	8.2	Ertugliflozin 5 or 15 mg/d	Placebo	42	MedDRA Preferred Term for AKI
Zannad et al. (2021) no CKD	R, DB, PC	Patients with HFrEF and no CKD	1746	63.9	77.9	79	92.5	75.2	Empagliflozin 10 mg/d	Placebo	16	MedDRA Preferred Term for AKI
Zannad et al. (2021) CKD	R, DB, PC	Patients with HFrEF and CKD	1978	70.2	74.5	46.9	86.8	67.8	Empagliflozin 10 mg/d	Placebo	16	MedDRA Preferred Term for AKI
Kosiborod et al. (2021)	R, DB, PC	Hospitalized patients with COVID-19 and at least one CV risk factor	1,250	61.4	57.4	83.8	35.5	NR	Dapagliflozin 10 mg/d	Placebo	3	MedDRA Preferred Term for AKI
Anker et al. (2021)	R, DB, PC	Patients with HFpEF	5,988	71.8	55.4	60.6	80.7	37.5	Empagliflozin 10 mg/d	Placebo	26.2	MedDRA Preferred Term for AKI
Bhatt et al. (2021a)	R, DB, PC	T2DM patients with CKD (eGFR: 25–60 mL/min/1.73 m^2^)	10,584	69	55.1	44.6	88.5	15	Sotagliflozin 200 or 400 mg/d	Placebo	16	MedDRA Preferred Term for AKI
Bhatt et al. (2021b)	R, DB, PC	T2DM patients hospitalized for recent worsening HF	1,222	69.5	66.2	49.8	91.3	64.5	Sotagliflozin 200 or 400 mg/d	Placebo	9	MedDRA Preferred Term for AKI
Solomon et al. (2022)	R, DB, PC	Patients with HFpEF	6,263	71.7	56.1	61	77.7	42.6	Dapagliflozin 10 mg/d	Placebo	27.6	MedDRA Preferred Term for AKI
Voors et al. (2022)	R, DB, PC	Patients with ADHF	530	70.5	66.2	52	70	52	Empagliflozin 10 mg/d	Placebo	3	MedDRA Preferred Term for AKI
Feitosa et al. (2023)	R, OL	T2DM patients undergoing PCI	42	64.5	69	65.1	88.1	NR	Empagliflozin 25 mg/d	Blank	1	KDIGO criteria
EMPA-kidney 2023	R, DB, PC	Patients with CKD	6,609	63.9	66.8	37.3	85.1	7	Empagliflozin 10 mg/d	Placebo	24	An increase in SCr to 1.5-times a recent historical value or initiation of RRT
Cox et al. (2024)	R, OL	Patients with ADHF	238	64.5	61	52.F	52	50.5	Dapagliflozin 10 mg/d	Blank	1	MedDRA Preferred Term for AKI
Butler et al. (2024)	R, DB, PC	Patients after AMI	6,522	63.6	75.1	77.8	72.5	39.5	Empagliflozin 10 mg/d	Placebo	17.9	MedDRA Preferred Term for AKI

RAASi, renin-angiotensin-aldosterone system inhibitors; eGFR, estimated glomerular filtration rate; MRA, mineralocorticoid receptor antagonists; AKI, acute kidney injury; SGLT2i: Sodium/glucose cotransporter-2 inhibitors; R, randomized; DB, double-blind; PC, placebo-control; OL, open-label; T2DM, type 2 diabetes mellitus; CKD, chronic kidney disease; CV, cardiovascular; ASCVD, atherosclerotic cardiovascular diseases; HF, heart failure; ADHF, acute decompensated heart failure; HFpEF, heart failure with preserved ejection fraction; HFrEF, heart failure with reduced ejection fraction; AMI, acute myocardial infarction; COVID-19, Coronavirus Disease 19; NR, not reported; MedDRA, the Medical Dictionary for Regulatory Activities; PCI, percutanous coronary intervention; SCr, serum creatinine; KDIGO, kidney disease improving global outcomes; RRT, renal replacement therapy.

**TABLE 2:** Study quality evaluation via the Cochrane Risk of Bias Tool.

Study	Random sequence generation	Allocation concealment	Blinding in performance	Blinding in outcome detection	Incomplete outcome data	Reporting bias	Other bias
Zinman et al. (2015)	Low risk	Low risk	Low risk	Low risk	Low risk	Low risk	Low risk
Neal et al. (2017)	Low risk	Low risk	Low risk	Low risk	Low risk	Low risk	Low risk
Perkovic et al. (2019)	Low risk	Low risk	Low risk	Low risk	Low risk	Low risk	Low risk
McMurray et al. (2019)	Low risk	Low risk	Low risk	Low risk	Low risk	Low risk	Low risk
Wiviott et al. (2019)	Low risk	Low risk	Low risk	Low risk	Low risk	Low risk	Low risk
Heerspink et al. (2020)	Low risk	Low risk	Low risk	Low risk	Low risk	Low risk	Low risk
Cannon et al. (2020)	Low risk	Low risk	Low risk	Low risk	Low risk	Low risk	Low risk
Zannad et al. (2021)	Low risk	Low risk	Low risk	Low risk	Low risk	Low risk	Low risk
Kosiborod et al. (2021)	Low risk	Low risk	Low risk	Low risk	Low risk	Low risk	Low risk
Anker et al. (2021)	Low risk	Low risk	Low risk	Low risk	Low risk	Low risk	Low risk
Bhatt et al. (2021a)	Low risk	Low risk	Low risk	Low risk	Low risk	Low risk	Low risk
Bhatt et al. (2021b)	Low risk	Low risk	Low risk	Low risk	Low risk	Low risk	Low risk
Solomon et al. (2022)	Low risk	Low risk	Low risk	Low risk	Low risk	Low risk	Low risk
Voors et al. (2022)	Low risk	Low risk	Low risk	Low risk	Low risk	Low risk	Low risk
Feitosa et al. (2023)	Unclear	Unclear	High risk	High risk	Low risk	Low risk	Low risk
EMPA-kidney 2023	Low risk	Low risk	Low risk	Low risk	Low risk	Low risk	Low risk
Cox et al. (2024)	Unclear	Low risk	High risk	High risk	Low risk	Low risk	Low risk
Butler et al. (2024)	Low risk	Low risk	Low risk	Low risk	Low risk	Low risk	Low risk

### Meta-analysis results

**FIGURE 2:**
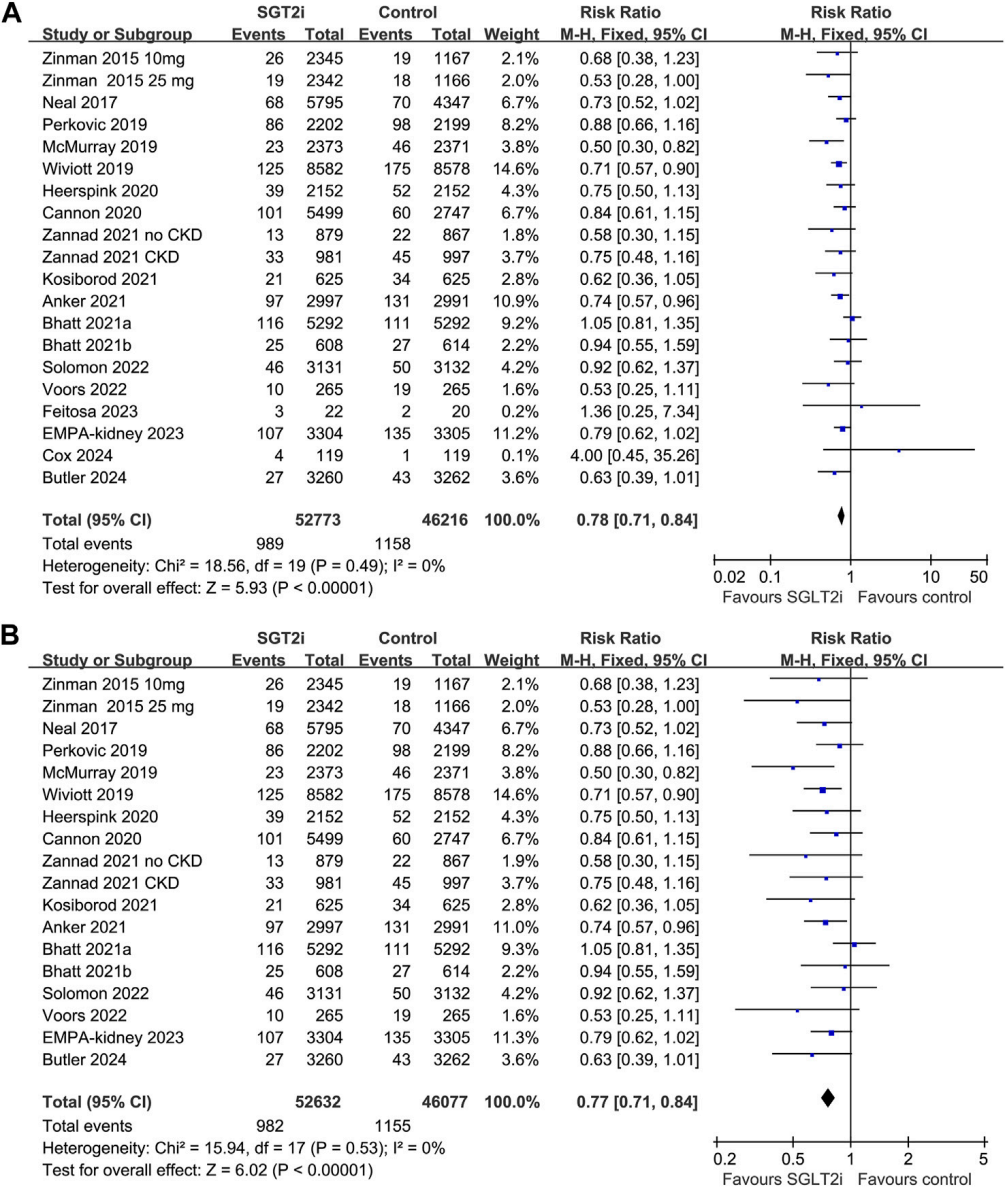
Forest plots for the meta-analysis of the influence of SGLT2i on the risk of AKI in adult patients; **(A)** the overall meta-analysis; and **(B)** the sensitivity analysis limited to high-quality studies.

**FIGURE 3:**
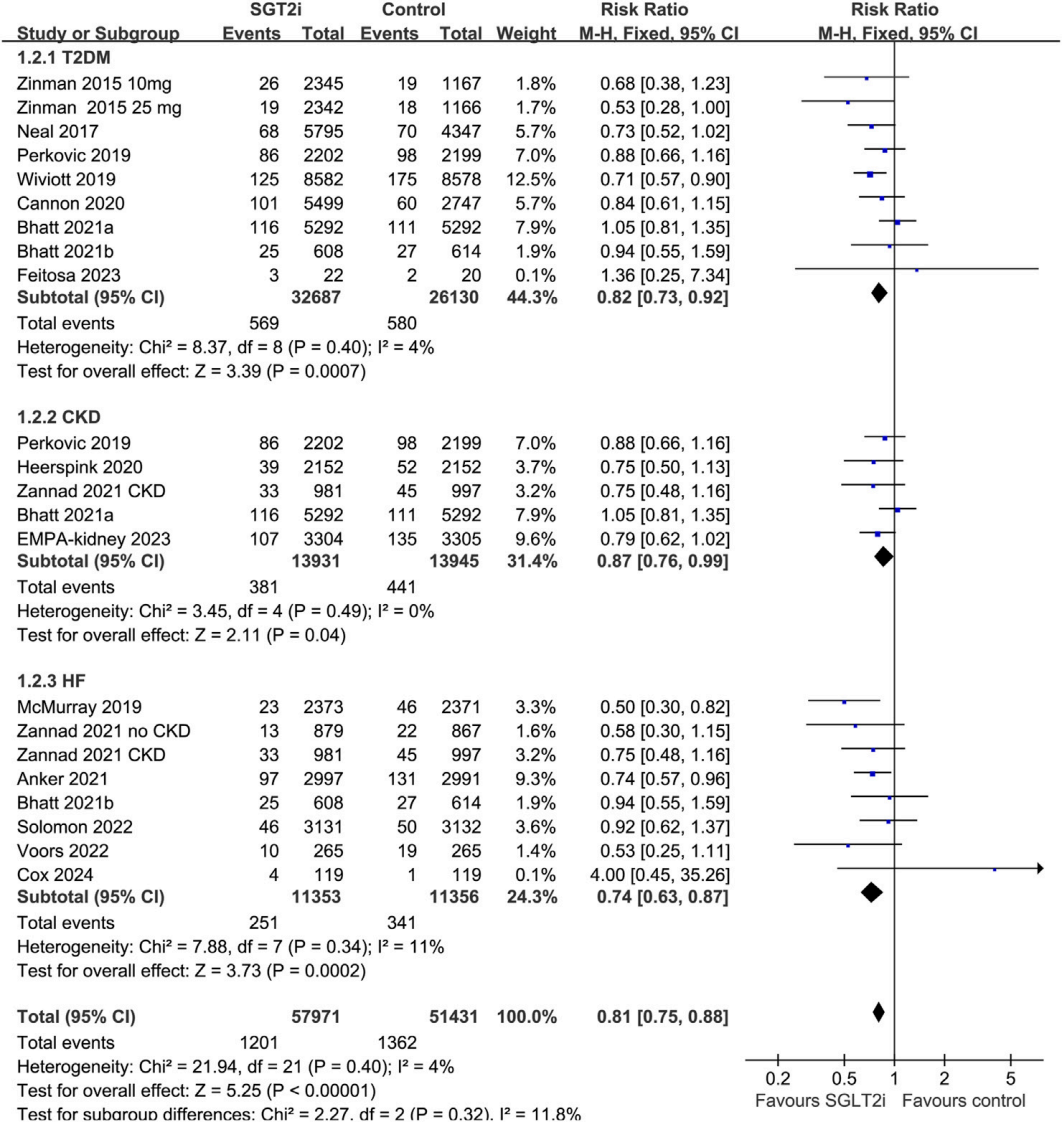
Forest plots for the subgroup analysis of the influence of SGLT2i on the risk of AKI according to the diagnosis of the patients.

**FIGURE 4:**
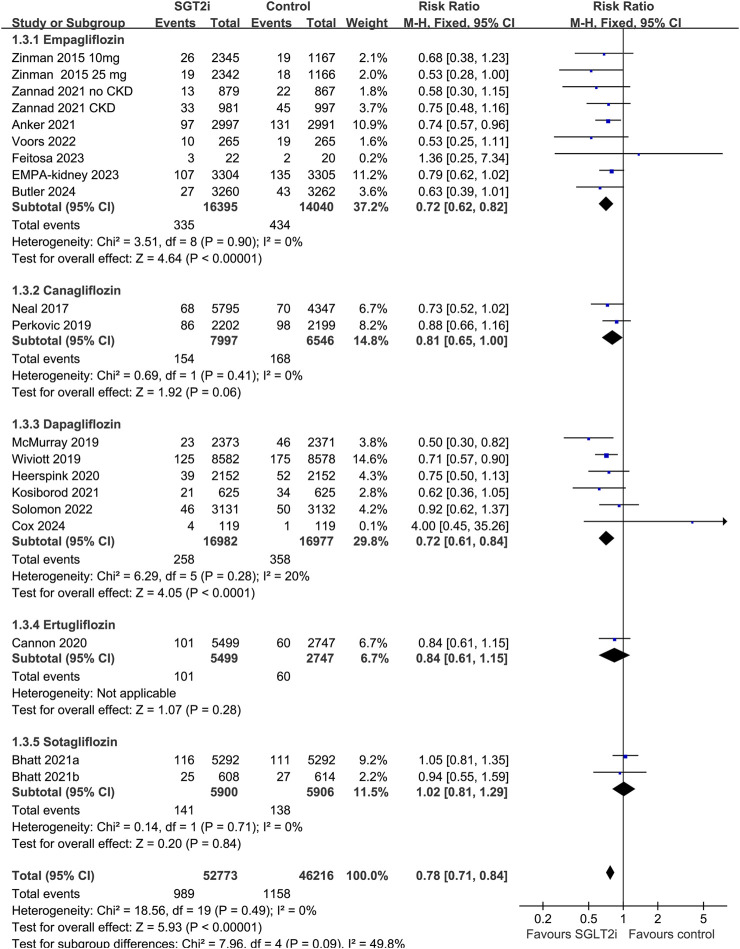
Forest plots for the subgroup analysis of the influence of SGLT2i on the risk of AKI according to individual SGLT2i drugs used.

Overall, 20 datasets from 18 RCTs, involving 52,773 patients receiving SGLT2i and 46,216 patients receiving placebo/blank treatment, were included in the meta-analysis. All the RRs and 95% CI were extracted from the original studies except data for one study (Anker et al., 2021), which was extracted from a previous meta-analysis after being provided directly to the authors (2022). Compared with placebo/blank treatment, treatment with SGLT2i significantly reduced the risk of AKI (RR: 0.78, 95% CI: 0.71 to 0.84, *p* < 0.001; Figure 2A) with no significant heterogeneity (for Cochrane Q test, *p* = 0.49; *I* ^2^ = 0%). The sensitivity analysis limited to high-quality studies (Zinman et al., 2015; Neal et al., 2017; McMurray et al., 2019; Perkovic et al., 2019; Wiviott et al., 2019; Cannon et al., 2020; Heerspink et al., 2020; Bhatt et al., 2021a; Anker et al., 2021; Bhatt et al., 2021b; Kosiborod et al., 2021; Zannad et al., 2021; Solomon et al., 2022; Voors et al., 2022; Herrington et al., 2023; Butler et al., 2024) showed similar results (RR: 0.77, 95% CI: 0.71 to 0.84, *p* < 0.001; Figure 2B). Subgroup analysis suggested consistent results in patients with T2DM (RR: 0.82, 95% CI: 0.73 to 0.92, *p* < 0.001; *I* ^2^ = 4%), CKD (RR: 0.87, 95% CI: 0.76 to 0.99, *p* = 0.04; *I* ^2^ = 0%), and HF (RR: 0.74, 95% CI: 0.63 to 0.87, *p* < 0.001; *I* ^2^ = 11%; *p* for subgroup difference = 0.32; Figure 3). In addition, subgroup analysis also did not suggest that the results were significantly affected by individual SGLT2i drugs used (*p* for subgroup difference = 0.09; Figure 4). Finally, univariate meta-regression with a random-effects model suggested that the influence of SGLT2i on the risk of AKI was not significantly modified by study characteristics such as mean age of the patients, proportion of men, baseline mean eGFR, proportion of patients with concomitant use of RAASi, proportion of patients with concomitant use of MRA, or mean follow-up duration (*p* all > 0.05; Table 3).

**TABLE 3:** Univariate meta-regression analysis.

	RR for the influence of SGLT2i on AKI		
Covariate	Coefficient	95% CI	*p*
Mean age (years)	0.011	−0.015 to 0.037	0.39
Men (%)	−0.010	−0.024 to 0.003	0.13
Mean eGFR at baseline (ml/min/1.73 m^2^)	−0.0042	−0.0097 to 0.0014	0.13
Any RAASi (%)	0.0041	−0.0057 to 0.0114	0.49
MRA (%)	−0.0020	−0.0062 to 0.0023	0.34
Follow-up duration (months)	−0.0027	−0.0095 to 0.0041	0.41

RR, risk ratio; CI, confidence interval; SGLT2i, Sodium/glucose cotransporter-2 inhibitors; RAASi, renin-angiotensin-aldosterone system inhibitors; MRA, mineralocorticoid receptor antagonists; eGFR, estimated glomerular filtration rate; AKI, acute kidney injury.

### Publication bias

**FIGURE 5:**
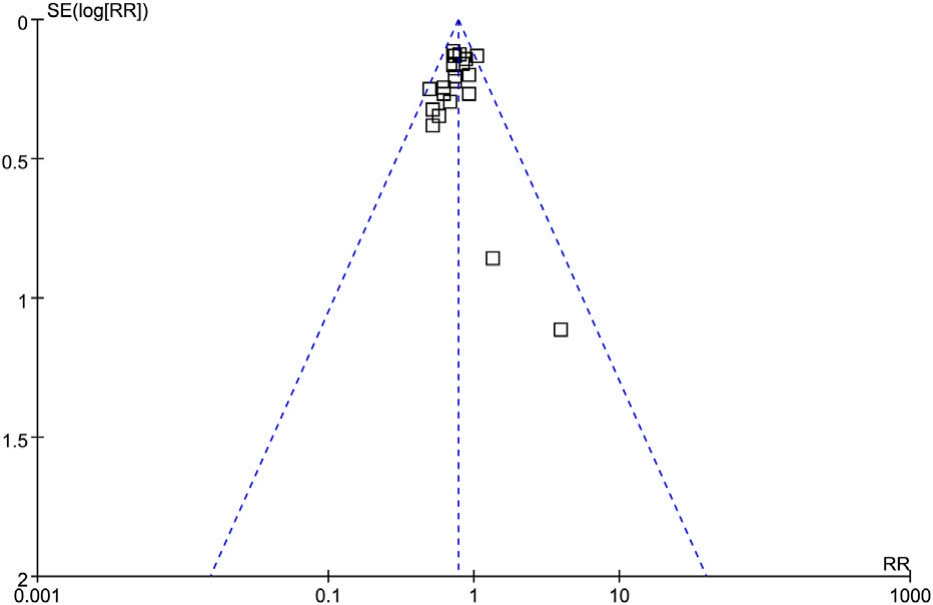
Funnel plots for the publication bias underlying the meta-analysis of the influence of SGLT2i on the risk of AKI in adult patients.

The symmetrical funnel plots observed in the meta-analyses of the impact of SGLT2i on AKI in adult patients indicate a minimal likelihood of publication bias (Figure 5). Furthermore, the results of Egger’s regression test support this notion, as it yielded a *p*-value of 0.32, indicating a low risk of publication bias.

## Discussion

In this meta-analysis, we conducted a comprehensive synthesis of data from 18 RCTs, comprising 20 datasets. The findings of our study indicate a significant reduction in the risk of AKI among adult patients when comparing the use of SGLT2i to placebo or blank treatment. The sensitivity analysis limited to high-quality RCTs showed similar results. Subgroup analyses further demonstrated consistent results in patients with T2DM, CKD, and HF. Additionally, our subgroup analysis suggests that the impact of SGLT2i on AKI does not appear to be influenced by the specific type of SGLT2i utilized. Finally, meta-regression analysis suggested that the influence of SGLT2i on AKI was also not likely to be modified by difference of study characteristics, such as mean age of the patients, proportion of men, mean baseline eGFR, proportions of patients with concomitant use of RAASi and MRA, or follow-up durations. Taken together, these results indicate that SGLT2i may be effective in reducing the risk of AKI, and the effect of SGLT2i on AKI may not be influenced by the baseline renal function or concurrent use of RAASi.

Some meta-analyses were published before or during the preparation this manuscript, which generally showed that SGLT2 inhibitors can exert the benefit in reducing AKI in patients with T2D, heart failure, or CKD; and this benefit does not vary with various characteristics, such as the diagnosis of the patients and type of SGLT2 inhibitors (Menne et al., 2019; Neuen et al., 2019; Zhao et al., 2020; Qiu et al., 2021; Baigent et al., 2022; Gong et al., 2022; Rigato et al., 2023). Compared to the previous meta-analyses, our study has several strengths. First, an extensive literature search was performed which retrieved 18 relevant up-to-date RCTs. Second, only RCTs were included, which minimized the biases related to the design of observational studies. In addition, although the results of the overall and subgroup analyses were generally consistent with the findings of the previous meta-analyses, we for the first time performed meta-regression analyses to investigate the potential influence of study characteristics such as age, baseline renal function, and concurrent use of RAASi at the study level. This is clinically important, because these factors have been related to the risk of AKI. Overall, results of the meta-analysis provided further evidence that SGLT2i may be effective in reducing the risk of AKI, and the effect might not be modified by age, baseline renal function and concurrent use of RAASi or MRA.

Although concerns have been raised reading AKI related to SGLT2i use in some case reports, subsequent investigations in high quality clinical trials and meta-analysis showed that SGLT2i may confer renal proactive efficacy and delay the deterioration of renal function (Lin et al., 2023). The current meta-analysis, by integrating the evidence from RCTs, further expanded the renal benefits of SGLT2i by showing that SGLT2i are effective in reducing the risk of AKI as compared to placebo/blank treatment. The potential mechanisms underlying the renal protective effect of SGLT2i may be multifactorial. An initial investigation conducted on non-diabetic mice using a renal ischemia/reperfusion injury model demonstrated that Luseogliflozin effectively mitigated peritubular capillary congestion/hemorrhage, alleviated hypoxia, and enhanced the expression of vascular endothelial growth factor (VEGF)-A, thereby exhibiting a protective effect on the kidneys during acute situations (Zhang et al., 2018). Furthermore, another study conducted on diabetic rats with myocardial infarction-associated AKI revealed that pretreatment with empagliflozin for 2 weeks resulted in improved hyperglycemia, elevated blood β-hydroxybutyrate levels, suppressed expression of NGAL and KIM-1 induced by MI, and ultimately prevented the pathogenesis of AKI (Kuno et al., 2020). Furthermore, previous research has demonstrated the significant reduction of both systemic and renal inflammation by empagliflozin, which has contributed to the observed survival benefits in an LPS-model of acute septic renal injury (Maayah et al., 2021). Additionally, a more recent study has indicated that dapagliflozin may mitigate contrast-induced acute kidney injury through the suppression of the hypoxia-inducible factor-1α pathway (Huang et al., 2022). Consequently, there is a need for further investigation into the key molecular pathways that underlie the preventive effectiveness of SGLT2i on AKI.

Results of subgroup analysis suggested that although no significant difference was observed for the influence of each individual SGLT2i drugs on AKI, the positive results were mainly driven by studies involving empagliflozin, canagliflozin, and dapagliflozin, but not for studies with ertugliflozin or sotagliflozin. However, these results should be interpreted with caution because only two datasets were available for the subgroups of ertugliflozin and sotagliflozin, and more studies are needed for further evaluation. Interestingly, results of meta-regression analysis suggested that the effect of SGLT2i on AKI did not seem to be significantly affected by eGFR at baseline, suggesting that potential renal protective efficacy of SGLT2i may also be consistent in patients with renal dysfunction before treatment (eGFR as low as 20 mL/min/1.73 m^2^). In addition, it has been suggested that excessive decline by SGLT2i combined with the excessive decline in trans-glomerular pressure induced by concomitant use of RAASi may further increase the risk of AKI (Szalat et al., 2018). Accordingly, we explored the influence of proportions of patients with concurrent use of RAASi and MRA on the effect of SGLT2i on AKI. Results suggested that the potential renal protective efficacy of SGLT2i may not be significantly modified by concurrent use of RAASi or MRA. This is consistent with a recently published *post hoc* analysis which showed that dapagliflozin consistently reduced the risk of kidney outcomes in T2DM patients irrespective of background use of various cardiovascular medications (Oyama et al., 2022). However, our results of meta-regression analysis according to baseline renal function and concurrent use of RAASi should be considered as exploring study because these results were based on the analysis of study-level data rather than individual-patient data.

This meta-analysis also has limitations. First, different SGLT2i drugs with different dosages were used among the included studies. Further studies are needed to determine if the influence of SGLT2i on AKI is consistent among individual SGLT2i drugs, and if there is a dose-effect relationship. Second, key aspects such as diabetes severity and duration, CKD, or HF, which have potential implications on SGLT2i effectiveness, may affect the influence of SGLT2i on AKI. Although, our meta-analysis is based on data at the study level rather than individual patient level; therefore, we were unable to determine the influence of these factors on the results. In addition, there are other medications which may also affect the risk of AKI besides RAASi and MRA, such as nonsteroidal anti-inflammatory drugs (NSAIDs). However, the status of NSAIDs use was generally not reported among the included studies, and we were therefore unable to determine its influence on the results of the meta-analysis. Moreover, we only included studies published in English as full-length article in peer-reviewed journals. Grey literature, such as conference abstracts and unpublished data were not included. Although excluding grey literature may improve the reliability of the finding because most grey literature are not strictly peer-reviewed, excluding these data may also increase the risk of publication bias. Finally, for most of the included studies, AKI was diagnosed based on MDRA preferred term for AKI. The influence of different diagnostic criteria for AKI, particularly those applicable in real-world clinical practice needs to be further evaluated.

As a summary, results of the meta-analysis suggest that SGLT2i may be effective in reducing the risk of AKI as compared to placebo/blank treatment in adult patients, and the influence of SGLT2i on AKI may not be affected by baseline renal function and concurrent use of RAASi.

## Data Availability

The original contributions presented in the study are included in the article/Supplementary Material, further inquiries can be directed to the corresponding author.
